# Therapeutic outcomes of mandibular advancement devices as an initial treatment modality for obstructive sleep apnea

**DOI:** 10.1097/MD.0000000000005265

**Published:** 2016-11-18

**Authors:** Pona Park, Hyoung Won Jeon, Doo Hee Han, Tae-Bin Won, Dong-Young Kim, Chae-Seo Rhee, Hyun Jik Kim

**Affiliations:** Department of Otorhinolaryngology, Seoul National University College of Medicine, Seoul National University Hospital, Seoul, Korea.

**Keywords:** initial treatment modality, mandibular advancement device, obstructive sleep apnea, therapeutic outcome

## Abstract

Although continuous positive airway pressure (CPAP) is a highly efficacious treatment for obstructive sleep apnea (OSA), there is a need for alternative treatment options, such as sleep surgeries and mandibular advancement devices (MADs), to overcome the limitations of CPAP.

This study aimed to analyze the therapeutic outcomes of OSA subjects who were treated with a MAD, and to estimate the clinical impact of MAD as a first-line treatment for OSA.

Forty-seven patients diagnosed with OSA received an adjustable MAD as an initial treatment. Drug-induced sleep endoscopic findings and sleep parameters (both pre-MAD and post-MAD treatment), such as apnea index, oxygen saturation, and degree of daytime sleepiness, were assessed retrospectively.

The MAD treatment resulted in a significant reduction in apnea–hypopnea index, and also a significant elevation in lowest oxygen saturation. Satisfactory results of MAD treatment as a first treatment modality were observed in 27 patients, and a successful outcome was reached in approximately 72% of patients. The OSA patients who had lower body mass index and upper airway narrowing at the level of palate and tongue base showed relatively higher rates of a satisfactory outcome even in cases of moderate or severe OSA.

These results suggest that the use of a MAD may be an alternative treatment option in OSA patients with retropalatal and retroglossal area narrowing regardless of disease severity. Additionally, MADs can be recommended as an initial treatment modality, and the effectiveness of MADs in achieving success may not be inferior to CPAP.

## 1 Introduction

Obstructive sleep apnea (OSA) is a common sleep disorder characterized by airway collapse at multiple levels of the upper airway, causing reduction or cessation of airflow during sleep. In the Starling resistor model, the upper airway is described as a hollow tube. Within this tube, reduced airflow from the nasal cavity and narrowing of the upper airway increases negative pressure in the pharyngeal airway and predisposes to pharyngeal collapse in a collapsible downstream segment.^[1]^ It has been reported that both upper airway narrowing and increased airway resistance may contribute to the underlying pathogenesis of OSA, leading to loud snoring, apnea, and systemic complications without proper treatment.^[2–6]^ Therefore, different therapeutic options have been proposed by researchers to improve upper airway narrowing and reduce airway resistance in OSA patients, including medical treatment and surgical interventions.

Various treatment modalities for OSA including behavioral modification, continuous positive airway pressure (CPAP), surgery, and oral appliances attempt to widen the upper airway and reduce airway collapsibility. Although CPAP appears to be most effective in improving sleep apnea, surgical correction of upper airway narrowing and oral appliances may be good alternatives for patients with mild to severe OSA who prefer to avoid CPAP or those who are unable to tolerate CPAP therapy.^[7,8]^ Mandibular advancement devices (MADs) are the most common class of oral appliances used to treat snoring or OSA and are considered simple, safe, and cost-effective options.^[9,10]^ All devices protrude the mandible and induce changes in the anterior position of the tongue, soft palate, and lateral pharyngeal wall, resulting in improved airway patency.^[11,12]^ Recent evidence has suggested that MADs also improve sleep-disordered breathing and are also typically used for OSA patients who demonstrate retroglossal area narrowing with mild OSA symptoms. In particular, MADs may be suggested to patients who have difficulty in tolerating CPAP treatment or are unable to undergo sleep surgery as an alternative treatment modality.^[9]^ MADs are classified according to adjustability when they contain a mechanism to increase the degree of mandibular advancement, or fixed when they are manufactured in a fixed position. They can be designed to monobloc or duobloc, depending on whether the MAD is composed of 1 or 2 pieces.^[13,14]^

The main advantages of MADs are the relative simplicity of the treatment and their reversibility in treating OSA. Based on the main objective sleep parameters, and also on changes in the upper airway, MAD might be effective to improve sleep architectures and sleep-related symptoms.^[15]^Although side-effects are frequently reported with MAD treatment, they are usually mild and acceptable.

However, the application of MADs has been limited to mild and moderate OSA patients as a supportive treatment method.^[9,10]^ Moreover, there are very few studies which objectively evaluate the effectiveness and therapeutic outcomes of MADs in the initial treatment of OSA despite satisfactory clinical outcomes with MADs.

The purpose of this study was to evaluate clinical outcome of MAD treatment for OSA patients when used as a first treatment modality. Furthermore, this study attempts to identify clinical or anatomic factors which are correlated with increasing the success rate of MADs.

## 2 Materials and methods

### 2.1 Patients and ethics statement

This study retrospectively reviewed the medical records of patients who were diagnosed with OSA by polysomnography (PSG) at Seoul National University Hospital from April 2012 to May 2015, including patients who were initially treated with an MAD. Patients using pharmacologic sleep aids, CPAP therapy, or those with a history of previous sleep surgeries were excluded from the study. A total of 47 patients were enrolled in the study. The study was approved by the Institutional Review Board at Seoul National University Hospital (IRB number 2015–0819). All patients in this study had both pretreatment and posttreatment full-night PSG performed, and also drug-induced sleep endoscopies before prescribing MAD.

### 2.2 Study design and sleep study

**Figure 1:**
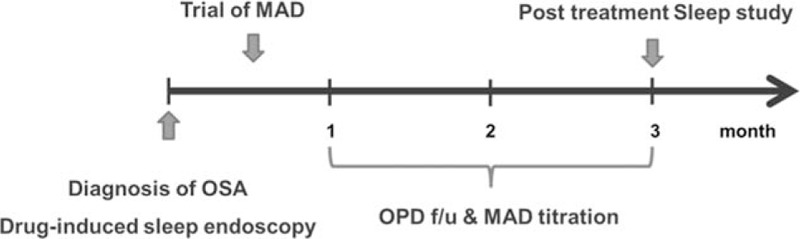
A schematic flow diagram for diagnosis of obstructive sleep apnea, drug-induced sleep endoscopy, MAD treatment, and follow-up studies. MAD = mandibular advancement device.

All subjects were required for having a pre and posttreatment sleep study to evaluate the intensity of snoring and OSA after MAD treatment. The sleep study performed in this study for full-night PSG was Neuvo (Compumedics, Victoria, Australia). C4/A1, C3/A2, O1/A2, and O2/A1 electrodes were used in electroencephalography, and 2 electrooculography electrodes were applied at the sides of both eyes to determine horizontal and vertical eye movements. Electromyography electrodes were attached to the submentalis muscles and both anterior tibialis muscles. A pulse oximeter was applied to the index finger to measure oxygen saturation, and nasal pressure cannulas were used to record airflow at upper airway. Strain gauges were used for recording chest and abdominal respiratory movements. The apnea–hypopnea index (AHI) identifies the number of apnea or hypopnea events per hour of sleep, and OSA is diagnosed in cases of 5 or more events identified on PSG. OSA severity is classified as mild (5 ≤ AHI < 15), moderate (15 ≤ AHI < 30), and severe (30 ≤ AHI). To evaluate the effect of MAD treatment, posttreatment PSG was performed 3 months after routine MAD usage (Fig. 1).

### 2.3 MAD

In this study, patients used monobloc-type MADs (Bioguard, Somnodent, Seoul, Korea). Alginate impressions of the patients’ mouths were taken and used to prepare study models. Maximum anterior movement was estimated and the bite was decided at the level of 60% of maximum anterior movement. In an attempt to minimize possible complications, including temporomandibular joint discomfort and constrained tooth movability, the MAD advancement was limited to less than 6.0 mm. A clear thermoforming plastic sheet (0.060 inch) was heated and compressed on the maxillary and mandibular casts with a vacuum instrument. The labial surface of teeth was covered at the level of one-third to half. Rough edges of casts were cut with a trimmer. The study models were applied to an articulator with the wax bite. Then, a monobloc device was made by plastering clear resin. We designed a minimal mouth opening because mouth opening inevitably rotated the mandible posteriorly and inferiorly. The treatment responses were classified into 4 categories as the change in AHI after MAD application: success, response, no response, and failure. Success was designated when AHI was reduced to greater than 50%, and less than 10 with MAD application. Response was designated when AHI was reduced to 20% to 50%, or reduced to greater than a 50% and an AHI of ≥10 with MAD application. No response was designated when AHI was reduced to less than 20%. Treatment failure was defined as an increased AHI after MAD application. Panoramic view and lateral cephalometric radiographs were obtained to evaluate the dental structure, temporomandibular joint, the skeletal patterns, and soft tissue abnormalities before MAD treatment. The Epworth Sleepiness Scale (ESS) was used to assess daytime sleepiness, and if ESS score was greater than 10, it was distinguished as daytime sleepiness. The therapeutic outcome of MAD treatment was assessed using AHI or oxygen saturation from PSG and questionnaires of ESS. All the patients’ data were collected by retrospectively analyzing medical records and PSG sleep parameters.

### 2.4 Drug-induced sleep endoscopy

Drug-induced sleep endoscopy (DISE) was performed in the subjects with supine position before attempting MAD, and heart rate and oxygen saturation were measured continuously throughout the procedure. Topical anesthesia was applied to the nasal cavity and decongestant cotton was placed at the middle meatus. Sleep was then induced with intravenous administration of midazolam (3 mg for adult patients over 50 kg; 0.06 mg/kg). After the patient fell asleep, endoscopic examination was carried out through the anesthetized nostril. The events for decrease of saturation (drop of basal saturation during sleep of more than 3%) were analyzed for obstruction level, structure, pattern, and degree, and representative findings were evaluated. If there was no decrease of saturation, changes during snoring were analyzed. Awakening events were controlled by administration of an additional bolus of 0.5 mg of midazolam, and the target level of sedation was muscle relaxation at upper airway producing obstruction (snoring or apnea) without respiratory depression. Midazolam-induced sleep endoscopic findings were classified according to the anatomic sites demonstrating obstruction (soft palate [SP], lateral pharyngeal wall including palatine tonsils [LW], tongue base [TB], and larynx including epiglottis [LX]). The degree of obstruction was scored as follows: 0 indicating no obstruction, 1 indicating partial obstruction (vibration with desaturation), and 2 indicating complete obstruction (total collapse of airway with desaturation). For scoring at tongue base narrowing, more than 50% displacement compared with the supine awake state was referred to as grade 1, whereas grade 2bstruction referred to more than 75% obstruction.

### 2.5 Statistical analysis

Continuous variables were presented as mean ± standard deviation (SD). The significance of differences between pre and posttreatment values of subjects who had MAD was evaluated by analysis of variance (ANOVA) with a post hoc test. Mann–Whitney *U* test was applied for the comparison of clinical and PSG or DISE parameters between the satisfactory and nonresponse groups. All analyses were performed with SPSS (version 18.0; SPSS Inc., Chicago, IL) for Windows software, and *P* values less than 0.05 were considered statistically significant.

## 3 Results

### 3.1 Clinical characteristics of subjects with MAD treatment

Forty-seven subjects diagnosed with OSA and prescribed MAD as an initial treatment for OSA were included in the present study, including 27 men and 20 women. The mean age of the subjects was 54.8 years and mean body mass index (BMI) was 24.6 kg/m^2^. Severity of OSA was classified based on AHI, and 13 subjects were classified as mild OSA, 17 as moderate OSA, and 17 as severe OSA.

The narrowing and collapsing sites within the upper airway were evaluated in subjects using DISE before MAD treatment. All patients showed grade 1 or 2 narrowing at the level of the tongue base. Airway collapse at the level of the soft palate was accompanied with tongue base narrowing in 21 patients (SP and TB). In addition, 10 patients were identified with tongue base obstruction, soft palate narrowing, and lateral collapse including tonsillar hypertrophy at once (SP, LW, and TB) (Table 1).

**Table 1:**
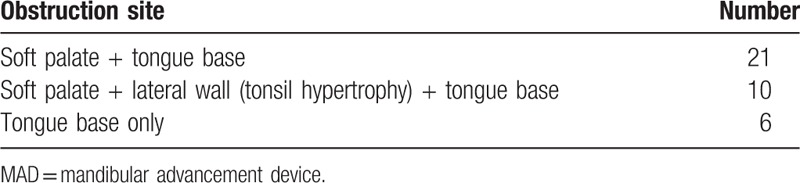
Obstruction sites of upper airway in subjects who were received MAD as first treatment modality.

### 3.2 Satisfactory outcome of MAD in the treatment of OSA

**Figure 2:**
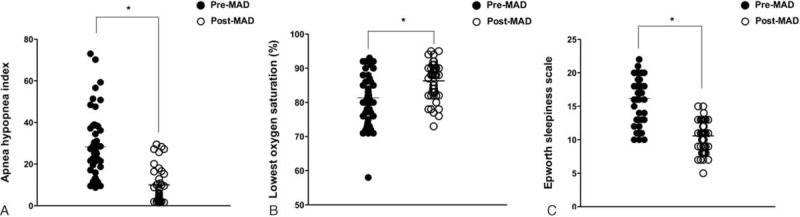
Changes in apnea–hypopnea index (AHI), lowest oxygen saturation, and Epworth sleepiness scale (ESS) after MAD treatment. (A) AHI and (B) lowest oxygen saturation were measured using full-polysomnography at baseline (pre-MAD treatment, n = 47) and follow-up after MAD treatment (n = 37). (C) ESS score was also examined before and after MAD treatment to assess the change in daytime sleepiness in the subjects (n = 47) who received MAD as an initial treatment modality (∗*P* < 0.05 comparing the values at pre-MAD treatment with those at follow-up after MAD treatment). MAD = mandibular advancement device.

In all patients, there were minimal technical complications and no complaints of adverse effects in the dentition or masticatory system as a result of wearing the MAD such as arthralgia, myofascial pain, or discomfort. Of the 47 patients, 10 patients who had MAD treatment as the first treatment modality did not attend a posttreatment check-up. Therefore, follow-up PSG was performed in 37 patients at 3 months after MAD treatment. At that time, we assessed the success rate of MAD treatment using AHI and the oxygen desaturation index of PSG. The AHIs for the patients both with and without the MADs are shown in Fig. 2A. The results showed a significant reduction in AHI among patients treated with MADs. In addition, the lowest oxygen saturation was significantly elevated up to 86.7%, and the ESS was considerably reduced after MAD treatment (Fig. 2B and C). Satisfactory results of MAD treatment as a first treatment modality were observed in 27 patients, and a successful outcome was reached in approximately 72% of patients. Sixteen patients were classified into the success group (greater than 50% reduction in AHI and an AHI <10). Eleven patients were classified into the response group (20%–50% reduction in AHI or greater than 50% reduction in AHI and AHI ≥10). However, MAD treatment was not effective for 10 subjects who were recommended MAD as a first-line treatment for OSA. Three of these no-response patients had surgical treatment instead of MAD application and 7 patients stopped using the MAD without any consideration for further treatment for OSA.

Through these findings, we found that MAD treatment would be effective for OSA patients, irrespective of OSA severity, and may be considered as a first-line treatment instead of CPAP or surgical treatment.

### 3.3 Clinical aspects of patients with satisfactory outcome using MAD treatment

We analyzed the clinical characteristics, DISE findings, and PSG results of the 27 patients who showed satisfactory outcomes (success and response group) after MAD treatment compared with patients who showed no response after MAD treatment.

**Figure 3:**
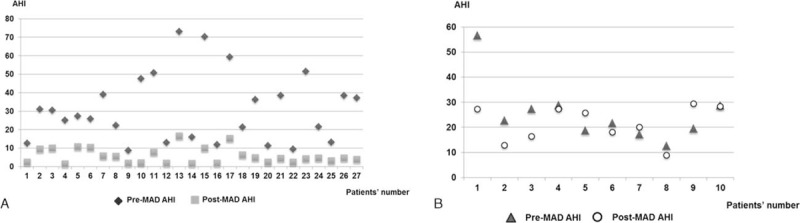
Apnea–hypopnea index (AHI) at baseline and at follow-up after MAD treatment in the satisfactory group and the no-response group. AHI at baseline and at follow-up after MAD treatment in the satisfactory group (AHI decreased by 50% in posttreatment MAD and AHI decreased below 20, n = 27) (A) and no-response group (n = 10) (B) was measured using full polysomnography. The value for each subject is shown and subjects are ordered by patient number. MAD = mandibular advancement device.

Both mean age and sex ratio were not significantly different between the 2 groups (satisfactory group vs no-response group). However, mean BMI of the satisfactory group was 22.91 kg/m^2^, significantly lower than that of the no-response group (28.01 kg/m^2^; *P* value = 0.03) (Table 2). AHI values at baseline and at follow-up after MAD treatment for members of the satisfactory group (n = 27) and the no-response group (n = 10) are shown in Fig. 3A and B.

**Table 2:**
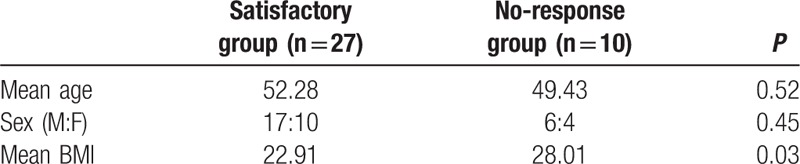
Baseline characteristics of the subjects in satisfactory and no-response group after MAD treatment (age, sex, body mass index [BMI]).

In the satisfactory group, 7 subjects were classified as having mild OSA, 8 having moderate OSA, and 12 having severe OSA. In the no-response group, 1 patient was classified as having mild OSA and 7 patients were classified as having moderate OSA (Table 3). We found that subjects with mild, moderate, and severe OSA were included in the satisfactory group, and the number of severe OSA patients was relatively higher in satisfactory group. We hypothesized that MAD treatment can be effective for OSA patients irrespective of OSA severity, and provides satisfactory results to OSA patients with a severe degree of sleep apnea.

**Table 3:**
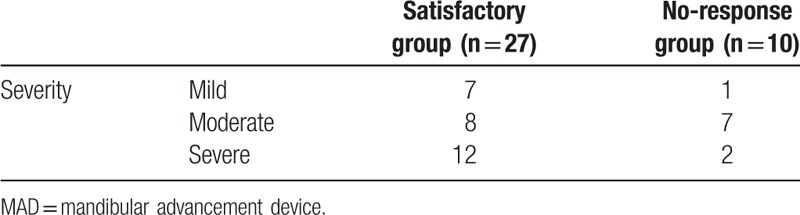
The distribution of disease severity in satisfactory and no-response group for MAD treatment.

We recommended MAD treatment as a first-line treatment for patients who showed tongue base obstruction. MAD treatment showed a higher probability of success among OSA patients with soft palate obstructions accompanied by tongue base obstruction. However, MAD treatment might not be effective as a first-line treatment for OSA patients with lateral wall obstruction and tongue base obstruction. If OSA patients had only tongue base obstruction, there was also significantly higher probability of satisfaction for OSA treatment using MAD (Table 4).

**Table 4:**
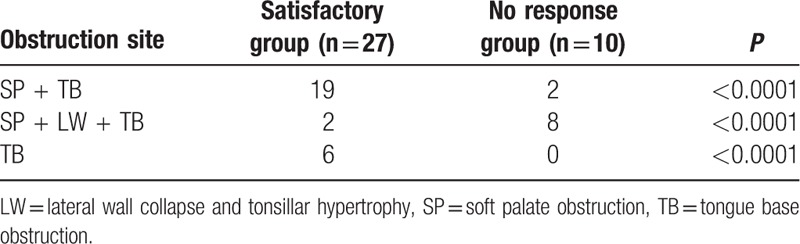
The comparison of drug-induced sleep endoscopic findings according to obstruction sites in satisfactory group and no response group of MAD treatment.

Based on these results, we suggest that MAD could be recommended for OSA patients as a first-line treatment modality, even among those classified as having severe OSA. Additionally, among patients with lower BMIs and soft palate obstructions with tongue base narrowing, there may be higher probability of successful MAD treatment.

## 4 Discussion

We found that MADs might be successful in the treatment of OSA as a first-line therapeutic modality, resulting in the improvement of several sleep-related variables. The current study proposes that MAD treatment could be preferentially recommended to OSA patients with tongue base narrowing and soft palate obstruction, and MADs have the potential to provide clinically satisfactory therapeutic outcomes in the reduction of upper airway narrowing.

The main pathologic conditions in OSA are upper airway collapse and narrowing, and many anatomical factors at the level of the pharynx may contribute to these pathologic conditions.^[6,16]^ Effective treatment of OSA requires maintenance of upper airway patency during sleep, which can be achieved through several modalities, including invasive and noninvasive interventions.^[17–25]^ For the treatment of OSA, there are several options from nonsurgical approaches such as weight control and intranasal space maintaining to surgical approaches including palatopharyngoplasty.^[26]^ CPAP is the current mainstay of noninvasive therapy for OSA and is an effective treatment that has been shown to reduce apnea or hypopnea, subjective sleep-related symptoms, and attenuate cardiovascular complications.^[3,27–29]^ Despite its clinical effectiveness and high success rate of CPAP for improvement of OSA, adherence to its use is poor and pressure intolerance is a frequent complaint reported by CPAP-nonadherent patients.^[30,31]^ As a nonsurgical approach, MADs are now widely recommended as an alternative treatment strategy for subjects who were diagnosed with mild or moderate OSA and who are unable to tolerate CPAP therapy.^[32–34]^ MADs had been actually used as a device for reducing snoring within the dental field and are often more tolerable for certain OSA subjects with appropriate oropharyngeal anatomy.^[34]^

In 2006, the American Academy of Sleep Medicine (AASM) updated the practice parameters for the treatment of OSA with oral appliances. They stated that oral appliances are indicated for use in mild-to-moderate OSA patients who prefer oral appliances to CPAP, do not respond to CPAP, are not appropriate candidates for CPAP, or have experienced failed treatment attempts with CPAP or behavioral measures such as weight loss and sleep position change.^[35]^ Some evidence suggests a significant improvement in symptoms and sleep study parameters through MAD application based on the correction of upper airway collapse at the retroglossal areas in OSA patients.^[36,37]^ However, oral appliance therapy has generally been thought to be less effective in relieving upper airway obstruction when compared with CPAP, and the positive impact beyond that observed in individual cases of MAD use as first treatment modality has not been systematically investigated.^[38]^ In addition, there has been controversy over the success rate of MAD treatment in controlling OSA, and large variability is observed in the reduction of AHI with MADs.

In the present study, we found that 72% of patients achieved a satisfactory outcome with 50% or greater AHI reduction, and an AHI of less than 10 per hour after MAD treatment. We also observed that satisfactory outcomes could be achieved in OSA patients irrespective of OSA severity, and 37% of subjects who showed a satisfactory outcome had severe OSA.

It has been reported that MAD treatment is less efficacious than CPAP for improving the sleep parameters of OSA. Only a few studies have reported a therapeutic effect of MAD treatment for the control of OSA in patients with mild sleep-related symptoms.^[39–41]^ Previous studies also have estimated that patients with severe OSA treated with MADs have shown lower success rates, and the results showed the evidence of efficacy of MAD for OSA patients and total success rate of MAD was below 50%.^[32,33]^ Accordingly, MADs have not been recommended as an initial treatment for patients with moderate or severe OSA.

However, we determined that MADs could be recommended to OSA patients with more extensive symptoms including moderate or severe OSA. Our study also suggested that MADs may not be inferior to CPAP in achieving successful outcomes and normalizing PSG indices in the treatment for OSA. Therefore, we concentrated on evaluating the clinical characteristics of patients who benefitted from MAD treatments irrespective of OSA severity. Future studies may be useful for identifying sleep parameters or anatomic structures of patients who do benefit from MAD treatment.

Based on the current findings, patients with severe OSA received pre and posttreatment PSG, and successful response to treatment was achieved in approximately 83% in the present study. In addition, a significant decrease in AHI with MAD treatment was observed in patients with moderate OSA, and the reduction in AHI reached 50%, indicating successful MAD treatment. We propose that MADs also showed a higher success rate in the treatment of patients with moderate or severe OSA, and this success rate is comparable with rates reported in the few recent studies on MAD treatment of severe OSA.

We also found that among patients with lower BMIs, OSA treatment with MADs resulted in a greater degree of improvement in sleep parameters and sleep quality. Besides severity of OSA and patients’ BMI, the exact evaluation for the level of airway narrowing might be critical to predict success rate of MAD treatment. We assessed the upper airway at the retropalatal and lateral pharyngeal wall, and examined for tonsillar hypertrophy and tongue base levels using sleep endoscopy. The effectiveness of MAD treatment was significantly higher for OSA patients with airway narrowing or increased collapsibility at both the soft palate and tongue base or the tongue base level alone. We determined that OSA patients with narrowed or collapsed airway at the retropalatal and retroglossal levels could expect significant improvement in AHI and oxygen saturation with MAD treatment. Thus, sleep endoscopy is essential to assess upper airway narrowing before prescribing an MAD.

Although the present study suggests the possibility of MAD as a more effective and potent treatment modality for moderate and severe OSA, the limitations of this study are several, and maybe the largest one is that we did not obtain adequate data related to subjective outcome of the MAD treatment from large population. In addition, most of the DISE findings had large range of variance in this study and it was suspected that there were individual differences in obstructive pattern at upper airway. Therefore, more large-scaled clinical study should be designed for the further analysis of MAD treatment including pre and posttreatment PSG and DISE findings to assess the effectiveness of MAD as a first treatment modality against moderate or severe OSA.

However, our results for therapeutic outcome of MAD are very promising, especially for severe OSA patients, who are at the greatest risk for serious medical consequences if untreated. Initial treatment of OSA with MADs can provide satisfactory results by reducing sleep variables, sleep apnea, and snoring, irrespective of disease severity, and the degree of improvement is dependent on patient-specific anatomic variables. Assessment of upper airway narrowing would be helpful to elevate the potential success rate of MAD treatment and to determine the precise indications for MAD treatment among OSA patients.

We concluded that MAD treatment could be preferentially recommended to OSA patients with tongue base narrowing and soft palate obstruction, and has the potential to provide clinically satisfactory therapeutic outcomes in the treatment of OSA irrespective of disease severity.
